# Research on the Paths and Effectiveness of Governance Tools Based on the Evolution of Environmental NIMBY Event

**DOI:** 10.3390/ijerph19041985

**Published:** 2022-02-10

**Authors:** Peng Xu, Xinyue Yao, Lan Lan, Ke Xu, Cunkuan Bao

**Affiliations:** Department of Environment Science and Engineering, Fudan University, Shanghai 200438, China; 19110740043@fudan.edu.cn (P.X.); yaoxinyue@pric.org.cn (X.Y.); 17110740040@fudan.edu.cn (L.L.); 19210740074@fudan.edu.cn (K.X.)

**Keywords:** environmental NIMBY events, governance tools, effectiveness, mediating effect

## Abstract

Identifying the path and effectiveness of governance tools is the key to environmental NIMBY (not in my back yard) event governance. However, there are limited studies on the path between effective governance tools and environmental NIMBY events. Based on the theory of emotional catharsis, we establish an analytical framework for the evolution of the environmental NIMBY event and analyze the effectiveness of the current main governance tools. The results show that government solicitation of opinions (GSOs) governance tools are insignificant in the governance of resistance behavior. The effects of public demand communication (PDC) governance tools and compensation negotiation (CN)governance tools on resistance behaviors all show a significant negative correlation; negative emotions play an intermediary role in their governance tools. The overall performance is that the greater the compensation, the lower the willingness to engage in resistance behavior. The establishment of a reasonable compensation system can effectively reduce the public’s willingness to engage in resistance behavior. Through the evaluation of the effectiveness of governance tools in environmental NIMBY events, this study helps to improve governance tools and has important practical significance for solving the environmental NIMBY dilemma.

## 1. Introduction

With the increase of population density in urban space and the improvement of public living standards, the public’s demand for environmental NIMBY facilities is increasing [1]. However, the new environmental NIMBY facilities, such as waste treatment and disposal facilities, sewage treatment plants and substations, will cause negative emotions among the neighboring residents, triggering opposition and resistance from the residents and forming an environmental NIMBY event. The environmental NIMBY event is a problem that must be faced by urban environmental management [2]. In particular, the rise of online media has provided a platform for the dissemination of environmental NIMBY events, making the impact of an environmental NIMBY event go beyond the geographical area affected by environmental NIMBY facilities [3,4,5]. The resistant public support and the large-scale diffusion of the NIMBY problem transfer the negative externalities derived from the NIMBY problem to the government itself, which has a serious negative impact on the image and credibility of the government.

Since the 18th CPC National Congress, the Chinese government has formulated, revised, and improved a large number of laws, regulations, rules, and systems, and the governance of environmental NIMBY events has shifted from “reactive governance” to “proactive management“ [6]. The government has phased out the confrontational approach to govern environmental NIMBY events and has put the perspective of governance before the event, such as establishing public participation mechanisms, including providing compensation. However, China’s environmental NIMBY events are becoming a social phenomenon of risk normalization, and “governance failures” still occur [7]. China is in a period of social transition, and the governance of environmental NIMBY events is related to social stability. How can governance tools resolve China’s environmental NIMBY events? How effective are various governance tools in environmental NIMBY event governance? The answers to the above questions will undoubtedly help improve governance tools and have important practical significance in solving the environmental NIMBY dilemma in China.

## 2. Literature Review

“NIMBY” (not in my back yard) usually refers to things with negative externalities and damaging ecological value [8,9]. Tracing back to the phenomenon of NIMBY, it is the negative emotion and resistance behavior caused by the residents near the facility affording the additional negative external cost of NIMBY facilities [10]. The environmental NIMBY event refers to the behavior where residents use violent or non-violent means to obstruct and protest the environmental NIMBY facilities or construction plans promoted by the government in order to keep the original living space from being broken, the living state from being disturbed, and the living environment from being polluted [11,12]. From the perspective of government decision-making, people’s consciousness of “right-safeguarding” and the subject consciousness of decision-making participation have achieved a new high level, but some grass-roots governments are not fully aware of this change [13]. Some scholars found that the grass-roots government followed the principle of collective discussion in the decision-making process of NIMBY projects, but the decision of collective discussion did not fully reflect the core demands of multiple decision-makers; this is an important inducement factor for the expansion of the NIMBY problem [14,15]. 

The existing research of environmental NIMBY events can be divided into four perspectives: emotional change, interest game, information dissemination, and conflict response. Liu made a survey of 2500 residents in China; the result shows that the awareness of civil rights increases with the upgrading of economic level, and the public’s aversion to NIMBY facilities is more than fear. Liu states that the cause of NIMBY conflict is that the proximate residents afford the expected loss caused by the adverse impact of NIMBY facilities. Compensation can reduce the occurrence of conflict but cannot completely avoid conflict [16]. Wei studied the emotional changes of the proximate residents caused by the new chemical plant using the structural equation model (SEM); the results show that the sense of unfairness and dissatisfaction with the government will increase the probability of environmental NIMBY events [17]. In addition, direct or indirect factors such as people’s risk perception [18,19], differential government trust [2], psychological acceptance [20], and the “emotional state” of proximate residents [21] will increase the probability of public resistance. The difference between the significant positive externalities of the NIMBY facilities to the grass-roots government and the negative externalities relative to the residents near the NIMBY facilities leads to the imbalance of benefit distribution [22]. Therefore, some scholars have studied how to design a more scientific compensation for the negative externalities afforded by the residents near the NIMBY facilities, such as the combination of compensation methods [23] and the grasp of compensation opportunities [24].

The information dissemination mainly discusses the dissemination path of NIMBY events. Rong studied the occurrence and spread of 150 large environmental NIMBY events from 2003 to 2014. They found that the network exposure of environmental NIMBY events will lead to the decline of government credibility, and the spread of rumors will greatly interfere with public judgment, thus causing environmental NIMBY events. The duration of events is concentrated within 2 weeks [25]. Xifra states that social networking, such as Weibo weblogs, plays a role in persuasion and information dissemination in NIMBY events [26]. The spread of false information is the main cause of network public opinion. The conflict response refers to the government’s response. As an important subject of NIMBY projects, the government plays a role in construction and operation [27]. It mainly acts on conflict response through administrative ideas and administrative actions. This research mainly involves government public opinion guidance [28], the role of government behavior on public space rights and interests [29], and the government’s attitude towards public protest issues and internal differences of opinion [30]. 

The existing research has studied the environmental NIMBY event from many angles, but few have discussed governance tools to change the path of public behavior and the effectiveness of governance tools. The effectiveness of governance tools is related to the promotion of NIMBY projects, and the impact or disputes generated by governance tools will even exceed the NIMBY projects themselves, which is directly related to the smooth implementation of the projects. Bruce Dorn points out that the choice of governance tools depends on the preferences of decision-makers and the response of social subjects to governance tools [31]. However, most of the existing studies focus on the public’s risk perception and attitude towards NIMBY facilities, ignoring the regulatory role of governance tools. Only a few studies on governance tools have focused on public participation, ignoring the correlation between different governance tools and the lack of in-depth and comprehensively logical combing of the dynamic evolution process of governance tools’ perception of public psychology, and they rarely describe the dynamic change process of the impact of governance tools on the public, from the construction to the operation of NIMBY facilities. Resistance behavior is a way for people to get psychological comfort by venting their negative emotions, and the governance tool is to defuse public anger and avoid resistance behavior. Therefore, based on the theory of emotional catharsis, this paper combines the logic of the development of environmental NIMBY events with people’s emotional changes, constructs a theoretical analysis framework of the effectiveness factors of environmental NIMBY event management tools, analyzes the effectiveness and relevance of different governance tools, and puts forward improvement schemes to improve the governance ability of environmental NIMBY events.

## 3. Materials and Methods

### 3.1. Analysis of the Effect of Emotional Catharsis in the Evolution of Environment NIMBY Events

Emotional catharsis utility refers to the psychological utility obtained when people’s negative emotions are vented in some way. In the process of environment NIMBY events, the development and change of people’s emotional catharsis effect can be divided into two stages: 

The first stage is project announcement. When the local residents involved find out about the NIMBY facilities, they will express their dissatisfaction with the government due to the negative impact of the NIMBY facilities. The negative impact on the local residents is caused by psychological damage and actual damage. Psychological damage includes a sense of psychological imbalance and risk anxiety as a victim [12]. The sense of psychological imbalance stems from the negative externalities brought by the NIMBY facilities, but the beneficiaries of the NIMBY facilities are the general public. The risk anxiety comes from the actual damage to the local residents after the completion of the NIMBY project. According to the previous cases of environmental NIMBY facilities, the existence of environmental NIMBY facilities will lead to the depreciation of the real estate around the facilities or increase the disease risk of the local residents. In the process of waiting for the government’s response, the discontent of the local residents shows a state of slow accumulation; the effect of emotional catharsis is gradually increasing, and the willingness of resistance behavior is increasing. In order to ensure the smooth implementation of the project, the government will carry out environmental impact assessments (EIAs), project hearings, and other means to solicit public opinions in order to promote the project.

The second stage is communication and negotiation. The project party and the local residents express their own demands, and the project party puts forward compensation to seek the continued promotion of the project. Finally, if the demands of local residents are not met, they may have negative emotions, such as anger and anxiety, and then take resistance behaviors, which will eventually lead to environmental NIMBY events. At this stage, the effect of emotional catharsis may far exceed the risk perception; especially in the scene of violent conflict, the people’s strong resistance to the government’s behavior plays an extremely important role in urging their resistance behavior.

There is a difference between people’s emotional catharsis utility and their risk perception. Governance tools will have an impact on people’s risk perception, but this risk perception value is relatively stable; it will not continue to expand or decrease over time, while the emotional catharsis utility will continue to change with different dissatisfaction. Therefore, the effect of the people’s emotional catharsis can provide a better explanation for the effectiveness of governance tools in terms of internal motivation.

### 3.2. Determinants and Hypotheses

**Figure 1:**
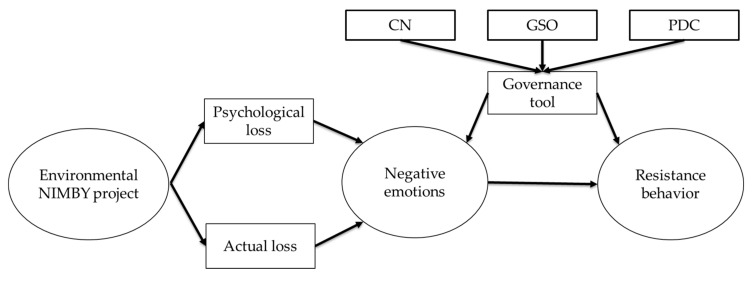
Theoretical analysis framework.

The effectiveness of governance tools depends on the response of social subjects to governance tools. Based on the interactive relationship between the government and the public, this study divides the governance tool in environmental NIMBY event governance into the government solicitation of opinions (GSO), public demand communication (PDC), and compensation negotiation (CN). GSO means that the government initiative takes information disclosure of the NIMBY project and understands the public’s knowledge of the NIMBY information. PDC refers to the process in which the public actively expresses their demands to the government to participate in NIMBY project decision-making. CN refers to the views of the government and the public on the negative externality compensation standard of NIMBY facilities. In the causal chain of public emotion and public behavior, the governance tool is an important external factor for the escalation or mitigation of NIMBY conflict. Effective governance tools can make NIMBY conflict controllable and benefit all participants in the event. Based on the above analysis of the evolution process of environmental NIMBY events, the effectiveness analysis framework of governance tools is shown in Figure 1.

#### 3.2.1. Government Solicitation of Opinions

It is not only the requirement of democratic governance but also the inevitable trend of social development to let the people with the most sensitivity to their own interests participate in the decision-making of NIMBY facilities. The project party (usually the local government) should build an agenda participation mechanism based on the public’s right to know. An EIA public participation and public representative hearing system is the main policy tool.

H1. The EIA public participation and hearing system has a negative impact on resistance behavior.

#### 3.2.2. Public Demand Communication

From the perspective of the public, in the process of safeguarding rights, the most important thing is whether their requirements can be effectively expressed and fed back. During the construction of NIMBY facilities, local residents lack effective ways to reflect their interest demands and opinions to relevant management departments due to interest damage and unfair treatment, which will produce negative emotions such as anger and anxiety. The feedback channel for public appeal expression is not smooth, resulting in the accumulation of negative emotions.

H2. Access to government information, communication platforms, and participation in policy decision-making has a negative impact on resistance behavior.

#### 3.2.3. Compensation Negotiation

The provision of NIMBY facilities is the responsibility of the government in order to improve public services, but it is also the responsibility of the government to overcome the negative externalities of such public facilities. Assuming that science and technology can not eliminate the negative externalities of NIMBY today, the only way to solve the imbalance of the policy interest structure is to rely on compensation policies. The NIMBY problem brings many losses to the economy, the environment, and health. It is necessary to formulate corresponding compensation policies for the losses.

H3. Economic compensation, environmental compensation, and health compensation have a negative impact on resistance behavior.

In addition, affected by individual characteristics such as income and qualifications, the public has a certain degree of self-regulation ability to the emotional impact brought by the environmental NIMBY project.

In this paper, the mediating effect model is adopted to verify the conclusion, with resistance behavior as the dependent variable, negative emotion as the independent variable, and governance tools as the mediating variable.

### 3.3. Selection of the Research Sample

In recent years, most NIMBY conflicts are mainly caused by waste incineration plants. Among the 52 large-scale environmental NIMBY events in the first half of 2016, 19 involved garbage, accounting for 36.5% [32]. 

The main data in this paper come from the investigation of the surrounding areas of the Shanghai JQ waste incineration plant. The JQ waste incineration plant was selected as the survey site because local residents had resisted the expansion of the waste incineration plant on the basis of the original site and had a clear understanding of environmental NIMBY events and strong representativeness. The field survey was conducted with a structured questionnaire. The subjects of the questionnaire are divided into the internal staff of the JQ waste incineration plant and local residents of 9 communities and villages within 3 km of the JQ waste incineration plant. The respondents were selected by random sampling. 

The main measurement items are shown in Table 1. All items are measured by a Likert level-5 scale. CN refers to the research of Zheng and is measured in three aspects: economic compensation, environmental compensation, and health compensation. In accordance with the relevant laws and administrative specification documents of China, environmental public facilities need to pass an EIA before project approval, so GSO refers to the measurement of the number of times the respondents have participated in the EIA and project hearings organized by the government. PDC is measured by the respondents’ understanding of appeal expression channels.

A total of 1092 questionnaires were collected, the invalid questionnaires were eliminated through the answer time and logic questions, and 1005 valid questionnaires remained. The reliability and validity of the sample data were tested. Table 2 presents descriptive statistics of the sample structure. From the results of the 1005 questionnaires, the proportion of men was 48.6% and that of women was 51.4%; The proportion of respondents under 18 years old was 11%, 39.8% were between 18 and 34 years old, 18.4% were between 35 and 44 years old, 13.3% were between 45 and 60 years old, and 17.4% were over 60 years old; 4% of the respondents have master’s degree or higher, 42.8% have received college and undergraduate education, 24.5% have received high school and technical secondary education, 22.9% have received junior middle school education, and only 5.9% have only received primary school education. In addition, individual characteristics also included the annual household income, nature of housing, and facility distance.

The Cronbach’s alpha coefficient of all variables was above 0.8, and the KMO (Kaiser-Meyer-Olkin) value of the validity index was 0.858. It can be considered that the construction of the questionnaire and its components is good.

## 4. Results

### 4.1. Analysis on the Action Path of Governance Tools on Resistance Behavior

The independent variable of this paper is resistance behavior, the dependent variable is GSO, PDC, and CN, and the intermediary variable is negative emotion. Negative emotions are mainly measured by the two dimensions of psychological loss and actual loss of the respondents, and the arithmetic average of the two is taken to measure the degree of negative emotions.

The mediating effect analysis in this paper refers to Wen’s research [33]; the results are shown in Table 3. On the basis of controlling for individual characteristics, the results in the first column of Table 3 were analyzed by binary logistic regression with governance tools as the independent variable and resistance behavior as the dependent variable. In the second column of Table 3, regression analysis was conducted with governance tools as the independent variable and negative emotions as the dependent variable. The results in the third column of Table 3 were analyzed by binary logistic regression with governance tools as the independent variable, negative emotions as the mediating variable, and resistance behavior as the dependent variable. In order to avoid multicollinearity in the regression results, the multicollinearity judgment method is used for testing. The VIF (variance inflation factor) was less than 10; that is, there is no multicollinearity between the independent variables.

### 4.2. Regression Result Analysis

#### 4.2.1. Influence of GSO on Resistance Behavior

The impact of public participation in EIA and hearing system on resistance behavior intention failed to pass the significance test, which may be due to the disconnection between these two governance tools and the establishment and management of NIMBY facilities. Some scholars point out that there is serious formalism in public participation in EIA and the hearing system [34,35]. Most of them are the “patching” practices of the government after the formation of decision-making schemes or even the implementation of projects, and their formal justice is greater than substantive justice. The impact of public participation in EIA on negative emotions passed the 5% negative significance test, and the hearing system on negative emotions failed to pass the significance test. It can be seen that compared with public participation in EIA, the hearing system is a more neglected governance tool. At present, the governance effectiveness of GSO cannot alleviate the NIMBY conflict but will bury hidden dangers for more intense conflicts.

#### 4.2.2. Influence of PDC on Resistance Behavior

The information channel passed the 5% negative significance test for resistance behavior. This suggests that the less information the public receives from the government, the more likely resistance will occur. In the process of the dissemination of NIMBY incidents, the negative reports on the network will enhance the confusion of NIMBY facilities, especially since the information related to the harm of NIMBY facilities is often mixed, including inflammatory remarks; reliable government information can avoid public panic. Communication platforms and decision-making channels did not pass the significance test at the initial stage, but under the mediation of negative emotions, their impact on resistance behavior intention passed the significance test of 10% and 5%. After adding negative emotion as a mediating variable, its coefficient decreased, indicating that the public’s negative emotion plays a significant mediating role between governance tools and resistance behavior.

#### 4.2.3. Influence of CN on Resistance Behavior

Environmental compensation and health compensation passed 5% and 1% negative significance tests on resistance behavior, respectively. Economic compensation, environmental compensation, and health compensation passed the significance test of 10%, 10%, and 1% for negative emotion, respectively. Economic compensation failed to pass the significance test at the initial stage, but under the mediation of negative emotion, the impact of economic compensation on resistance behavior intention passed the significance test of 10%. Under the mediating effect of negative emotion, environmental compensation and health compensation still passed the 10% significance test on the intention of resistance behavior, but their coefficient increased slightly after adding the mediating variable of negative emotion. It shows that the negative emotions of the interested people play a partial intermediary role in environmental compensation and health compensation. It is further confirmed that environmental compensation and health compensation can effectively control the negative emotions of the public and reduce the intention of resistance behavior.

## 5. Discussion

Through the above analysis of the action path and impact of governance tools on public resistance intention, among the three governance tools, CN is the most significant and GSO is the least significant. GSO does not play an effective governance role, which is consistent with the result that some scholars have pointed out—that public participation in EIAs and the hearing system of China’s environmental NIMBY projects is too formal [34]. From the side perspective of the project, although in relevant laws and regulations, public participation in EIAs and project hearings are regarded as an important part of the project establishment of NIMBY facilities; there is a lack of corresponding supervision and accountability mechanisms, and the project is not willing to implement this on the side. From the perspective of local residents, there is a lack of public knowledge about NIMBY projects—less than 200 of the 1005 respondents participated in the EIA or project hearing. The respondents who have participated in the survey generally reflect that it is difficult to put forward scientific opinions and make choices without knowing them. When the public is “represented”, it will distrust the NIMBY project, resulting in resistance when the public knows the environmental NIMBY information. Wang and Johnson’s research also believes that the lack of public opinion solicitation is an important factor in public resistance [32,35]. The environmental NIMBY facilities involve a wide range of public, complex interest relations and a complex structure of background factors such as identity and education level, and it is difficult to select representatives who truly represent the different categories of the public.

At present, the use of PDC governance tools can effectively alleviate the negative emotions of the public and reduce the probability of resistance behavior. In the early stage of NIMBY event development, conveying effective information to the public can reduce the probability of public resistance. Poor communication of demands will magnify the event and increase the voice channels of the residents involved, which can effectively reduce the amplification effect in the process of event fermentation. With the continuous development of NIMBY events, the timely building of a communication platform and allowing the public to participate in project decision-making will be the key to effective governance. However, during the questionnaire survey, most of the local residents did not know how to express their demands to the government, especially with the older people’s low utilization of the Internet and mobile phones. When their demands are not met, it is the last resort to participate in group resistance activities. In fact, the United States also experienced increasing environmental NIMBY events in the 1980s. Public demand communication is basically involved in most cases of the effective handling of environmental NIMBY events in the United States. The typical case is the site location of waste facilities in New York. The whole process of the site location of waste facilities go through the following steps: information publishing–feedback of resident-publicity of alternative plans–start construction and “urban land use approval procedures” as a supporting procedure [36]. Public demand communication improves the residents’ sense of identity to the project and reduces the resistance behavior of residents, which plays a positive role in effectively dealing with the occurrence of environmental NIMBY events.

CN is the most effective tool to control environmental NIMBY events. However, pure economic compensation cannot achieve the desired effect. Economic compensation cannot have a direct effect on resistance; the reasons can be divided into two aspects: on the one hand, most local residents have limited expectations of government compensation. They believe that there is no economic compensation in NIMBY facility construction or the economic compensation is limited, and the loss is far less than the actual loss. Some scholars have explained the compensation system. China has not yet formed effective and feasible policies and standards for the compensation of NIMBY facilities [37].

The concept of compensation of NIMBY facilities often appears in policy documents in the form of guidance, lacking relevant quantitative standards. Due to the lack of standards, extreme situations such as low compensation or excessive pricing can occur during compensation negotiations. At the same time, from the perspective of decision-making procedure, the project planning stage requires the design of a project compensation scheme; the project sideline may pay insufficient attention to the compensation scheme, especially ignoring the possible risks and benefits of the public. On the other hand, most of the public’s demands for health are far greater than money, especially for families with children.

## 6. Conclusions

Based on the survey data of the Shanghai JQ waste incineration plant, this paper uses the mediation effect model to explore the effectiveness of current governance tools for environmental NIMBY incidents. The results show that public participation in EIAs and hearing systems cannot effectively reduce the willingness of the public to produce resistance behaviors. The effects of PDC governance tools on resistance behaviors all show a significant negative correlation. Negative emotions play a significant mediating role in the governance of resistance behavior by government information and play a complete intermediary role in the governance of resistance behavior by communication platforms and decision-making channels. The CN is currently the most effective governance tool. Negative emotions play an intermediary role in the governance tools of CN. The overall performance is that the greater the compensation, the lower the willingness to engage in resistance behavior, and the establishment of a reasonable compensation system can effectively reduce the public’s willingness to engage in resistance behavior.

Based on the above conclusions, this paper puts forward the following suggestions:

Improve the quality of opinion solicitation, improve the public’s understanding of NIMBY facilities, ensure the public fully understands the project and effectively participates in the decision-making of the project, enable the public to fully express their interest preference, alleviate public anxiety, reduce negative emotions, and solve the public’s doubts and fears about the surrounding environmental projects from the source.

Set up the mechanism of the flow of communication to ensure that the public has unobstructed channels to express their demands. By building a convenient channel or platform for receiving and reflecting public appeal, the public will fully understand the project situation, and government agencies will be clearly fulfilling their responsibility by avoiding mutual shuffles that lead to negative public sentiment fermentation, nipping conflict resolution in the bud.

Improve the NIMBY compensation policy system, formulate guidelines or methods for NIMBY facility compensation, specify compensation standards applicable to different cities and regions, incorporate compensation into the planning stage of projects, and effectively protect the interests of the public affected by NIMBY projects.

Government departments should, at ordinary times, notice the media and experts in order to strengthen publicity and the education of the people, popularize scientific knowledge from the facilities, and improve public scientific literacy, knowledge levels, and the crisis response capacity. As far as possible, they should make the public resistance conscious of certain negative emotions, avoid the public’s fear of the unknown by engineering particular psychology, and reduce the promotion of adjacent projects when concerned about public feeling so as to reduce the possibility of environmental NIMBY events.

## Figures and Tables

**Table 1:** Measurement and design of variables.

Variable Name	Items	Value
Psychological loss (PL)	PL 1: Does negative news coverage worry you?	1–5 scale; 1 = strongly disagree, 5 = strongly agree
	PL 2: Does it worry you that environmental protection facilities such as waste incineration plants are harmful?	
	PL 3: Do you feel anxious about the unavoidable risks of environmental facilities such as waste incineration plants?	
	PL 4: Would it be unfair for waste incineration plants to be built near your home rather than somewhere else?	
Actual loss (AL)	AL 1: The construction of waste incineration plants around your home will cause health damage to yourself and your future generations	
	AL 2: The construction of waste incineration plants around your home will lead to the depreciation of accessory properties	
Compensation negotiation (CN)	CN 1: Are you willing to accept refuse incineration plants if reasonable financial compensation is provided?	
	CN 2: Would you agree to live near a NIMBY facility if there are schools, hospitals, parks, subway, shopping malls, or other public service facilities in the vicinity?	
	CN 3: Would you agree to live near a NIMBY facility if the government or enterprise regularly provides free physical examinations and psychological counseling services?	
Government solicitation of opinions (GSO)	GSO 1: Participated in the opinion solicitation in the EIA stage	1–5 scale; 1 = Never 5 = Always
	GSO 2: Participated in project hearings and demonstration meetings organized by the government	
Public demand communication (PDC)	PDC 1: There is access to government information	
	PDC 2: There is a platform for voicing concerns	
	PDC 3: There are ways to participate in and influence government decisions	
Resistance behavior (RB)	RB 1: If the government builds waste incineration plants, will you sign a joint letter against the project construction?	0–1 scale; 0 = No 1 = Yes
	RB 2: If the government builds waste incineration plants, will you spread negative information to other residents or the public through media, Internet, and other means to encourage other residents to resist?	
	RB 3: If the government builds waste incineration plants, will you participate in protests organized by others against the project?	
	RB 4: If the government builds waste incineration plants, will you take the initiative to initiate and organize other residents to boycott the project construction?	

**Table 2:** Demographic structure of the sample.

Attributes	S	*n*	Percent (%)
Gender	Male	488	48.6
	Female	517	51.4
Age	≤18	111	11.0
	18–34	400	39.8
	35–44	185	18.4
	45–60	134	13.3
	60	175	17.4
Level of education	primary school	59	5.9
	junior middle school	230	22.9
	high school and technical secondary education	246	24.5
	college and undergraduate education	430	42.8
	master’s or higher	40	4.0
Annual household income	≤100,000 yuan	400	39.8
	100,000–300,000 yuan	468	46.6
	300,000–600,000 yuan	105	10.4
	600,000 yuan	23	2.3
	≥1,000,000 yuan	9	9
Housing nature	Renter	276	27.5
	Resettlement house	207	20.6
	Commercial housing	502	50.0
	others	20	2.0
Facility distance	≤500 m	6	0.6
	500–1000 m	217	21.6
	1000–2000 m	360	35.8
	2000–3000 m	388	38.6
	≥3000 m	34	3.4

**Table 3:** Risk levels of various NIMBY facilities.

Variables	(1)	(2)	(3)
	Resistance Behavior	Negative Emotion	Resistance Behavior
GSO			
Public participation in EIA	−0.428	−0.263 **	−0.320
	(0.175)	(0.582)	(0.175)
Hearing system	−0.276	−0.086	−0.376
	(0.178)	(0.639)	(0.177)
PDC			
Government information	−0.896 **	−0.745 **	−0.146 *
	(0.129)	(0.077)	(0.130)
Communication platform	−0.215	−0.304 *	−0.178 *
	(0.120)	(0.431)	(0.120)
Decision-making channel	−0.660	−0.102 **	−0.518 **
	(0.124)	(0.488)	(0.124)
CN			
Economic compensation	−0.306	−0.445 *	−0.252 *
	(0.995)	(0.355)	(0.997)
Environmental compensation	−0.568 **	−0.719 *	−0.485 *
	(0.107)	(0.0391)	(0.107)
Health compensation	−0.191 ***	−0.250 ***	−0.110 ***
	(0.101)	(0.361)	(0.104)
Sex	0.505	0.337	−0.536
	(0.299)	(0.104)	(0.301)
Age	0.282	0.103	0.070
	(0.105)	(0.382)	(0.106)
Edu	−0.371 *	−0.132 **	−0.485 *
	(0.139)	(0.498)	(0.140)
Income	0.182 *	0.590 *	0.301 *
	(0.166)	(0.603)	(0.166)
Housing	0.383	−0.516	0.383
	(0.165)	(0.596)	(0.165)
Distance	−0.177 **	−0.348 *	−0.181 *
	(0.166)	(0.601)	(0.166)
Negative emotion			−0.790 ***
			(0.0873)
Constant	2.022 *	6.108 ***	2.516 **
	(1.184)	(0.438)	(1.309)
			
Observations	1005	1005	1005
R-squared	0.648	0.707	0.598

Standard errors in parentheses *** *p* < 0.01, ** *p* < 0.05, * *p* < 0.1.
